# Actions to mitigate ocular adverse events related to hair styling cosmetics in Brazil: a descriptive and correlational study (2022-2024)

**DOI:** 10.1590/S2237-96222024v34e20240449.en

**Published:** 2025-05-02

**Authors:** Daniel Marques Mota, Leonardo Oliveira Leitão, Ronald Santos Silva, Cássia de Fátima Rangel Fernandes, Marcus Tolentino Silva

**Affiliations:** 1 Agência Nacional de Vigilância Sanitária, Brasília, DF, Brazil; 2 Instituto Nacional de Controle de Qualidade em Saúde, Rio de Janeiro, RJ, Brazil; 3 Universidade de Brasília, Brasília, DF, Brazil

**Keywords:** Hair Preparations, Corneal Injuries, Product Surveillance, Postmarketing, Document Analysis, Correlation of Data, Preparaciones para el Cabello, Lesiones de la Cornea, Vigilancia de Productos Comercializados, Análisis de Documentos, Correlación de Datos

## Abstract

**Objective:**

To describe health surveillance actions implemented by the Brazilian Health Regulatory Agency (ANVISA) to mitigate cases of ocular adverse events related to hair styling creams in Brazil and verify correlation between reported cases and these actions.

**Methods:**

This is a descriptive and correlational study based on analysis of documents and the ANVISA case reporting database, between March 2022 and March 2024. Spearman’s correlation coefficient was used to examine correlation between ANVISA actions and cases of adverse events (p-value<0.05).

**Results:**

Three distinct periods were identified in relation to ANVISA actions (n=172) and reported cases (n=636). The second period (December 2022 to June 2023) presented the highest number of actions (n=125; 72.7%), coinciding with the peak of case reports (n=550). During this period, two safety alerts were published and a precautionary ban was ordered on all hair styling creams sold in Brazil, which lasted 38 days. In the third period (July 2023 to March 2024), the highest number of cream marketing cancellations occurred (n=3,122; 79.3%), and toxicology analyses indicated that six (30.0%) samples were classified as severely irritant/corrosive. There was a positive and strong correlation between the actions and the reported cases, ranging from 0.53 (p-value 0.006) to 0.74 (p-value<0.001).

**Conclusion:**

The strong positive correlation demonstrates that ANVISA’s actions were reactive to the increase in case reports, reinforcing the importance of active surveillance and rapid interventions to control risks to public health.

## Resumen

**Objetivo:**

Describir las acciones de vigilancia en salud implementadas por la Agencia Nacional de Vigilancia Sanitaria (ANVISA) para mitigar los casos de eventos adversos oculares relacionados con cremas para peinar el cabello en Brasil y verificar la correlación entre los casos notificados y esas acciones.

**Métodos:**

Estudio descriptivo y correlacional, basado en el análisis de documentos y de la base de datos de notificaciones de ANVISA, entre marzo de 2022 y marzo de 2024. Se utilizó el coeficiente de correlación de Spearman para examinar la correlación entre las acciones de ANVISA y los casos de eventos adversos (valor p<0,05).

**Resultados:**

Se identificaron tres períodos distintos en relación a las acciones de ANVISA (n=172) y a los casos notificados (n=636). El segundo período (diciembre de 2022 a junio de 2023) presentó el mayor número de acciones (n=125; 72,7%), coincidiendo con el pico de notificaciones de casos (n=550). Durante este período se publicaron dos alertas de seguridad y se ordenó una prohibición cautelar de todas las cremas para peinar el cabello comercializadas en el país, la cual duró 38 días. En el tercer período (julio de 2023 a marzo de 2024), se produjo el mayor número de cancelaciones de comercialización de cremas (n=3.122; 79,3%), y los análisis toxicológicos indicaron que seis (30,0%) muestras fueron clasificadas como irritantes/corrosivas severas. Hubo una correlación positiva y fuerte entre las acciones y los casos reportados, que oscilaron entre 0,53 (valor p 0,006) y 0,74 (valor p<0,001).

**Conclusión:**

La fuerte correlación positiva demuestra que las acciones de ANVISA fueron reactivas al aumento de las notificaciones de casos, reforzando la importancia de la vigilancia activa y de las intervenciones rápidas para controlar los riesgos para la salud pública.

## Introduction

Cosmetics safety has intensified in recent years, driven by increased consumption of these products (1), development of strict regulations (2), updates of banned chemical ingredients in cosmetics (3) and records of adverse events that affect consumers’ quality of life and burden healthcare systems (4-6). 

Most cosmetics are exempt from prior regulatory health authority assessment before being made available on the market (7), and it is up to the company to ensure that they are safe for health. This regulatory simplification also highlights the need for continuous and rigorous post-market surveillance to protect the population (7).

Post-marketing surveillance of cosmetics safety is known as cosmetovigilance. Since 2005, in Brazil, cosmetics manufacturing companies have been required by law to implement cosmetovigilance systems and report situations that the Brazilian Health Regulatory Agency (*Agência Nacional de Vigilância Sanitária* - ANVISA) deems to pose a health risk (8). Other countries have followed this trend, requiring companies to report serious cosmetics-related adverse events to health authorities (2). 

As of March 2022, in Brazil, hair styling creams began to be associated with risks to eye health, evidenced by reports of burning, pain, eye watering, irritation, hyperemia and, in extreme cases, corneal burns and temporary blindness (9-10). Cases increased in December 2022 (11), turning into a health crisis. 

The objective of this study was to describe the health surveillance actions implemented by ANVISA to mitigate cases of ocular adverse events related to hair styling creams in Brazil and to verify correlation between reported cases and these actions.

## Methods

### Design

This is a descriptive and correlational study, carried out based on analysis of documents produced or managed by ANVISA, including a database of reports of cases of ocular adverse events related to hair styling creams. The study covered the period from March 1, 2022 to March 31, 2024. The tacit knowledge (12) of one of the authors about ANVISA’s activities guided the delimitation of the period, definition of keywords and document sources.

### Setting

This study was based on health surveillance actions carried out by ANVISA, which regulates and monitors cosmetics to ensure their safety and effectiveness on the Brazilian market (13). ANVISA was selected as the focus of the study due to its central role in cosmetics regulation in Brazil, including implementing and monitoring actions to combat health crises; and also because it has produced a set of documents that offer a detailed view of the actions taken to mitigate adverse events related to cosmetics. 

### Definition of health surveillance action 

A “sanitary surveillance action” was defined as the production of documents issued by ANVISA or other entities, the creation of which was directly driven by the Agency as a result of ocular adverse events related to hair styling creams. These documents included field investigation and laboratory analysis reports and emails from international health authorities. This approach was based on the premise that these documents operationalized ANVISA’s sanitary surveillance actions.

### Inclusion and exclusion criteria

The inclusion criteria were: (i) documents that contributed to the study objective, such as normative acts, alerts, data sheets, news articles, bulletins, reports, letters and emails; (ii) documents on market inspection measures, marketing authorization procedures, cosmetovigilance actions, including field investigation and laboratory analysis; and (iii) documents generated between March 2022 and March 2024. 

The exclusion criteria included: (i) documents that corrected or revoked errors in previous documents; (ii) documents that canceled items from other documents without providing new information; (iii) documents not directly related to the topic; (iv) redundant or similar documents, in which only the first or most complete document was included, unless subsequent ones presented relevant new information; (v) documents with no issuing date; (vi) administrative procedure documents with no relevance to the study; and (vii) third-party response documents irrelevant to the study

### Data collection

The data sources were: the Federal Government Official Gazette of the Union; the ANVISA internet portal; the ANVISA Electronic Information System. Data collection took place between July and August 2024 (Table 1).

**Table 1:** Data sources, types of documents and search strategies for the documents used in this study

Data source	Type of document	Keywords	Selection filters	Internet URL
Official Gazette of the Union	Normative acts	Cera fixadora Cera modeladora Gel cera Gel cola Intoxicação ocular Pasta fixadora Pasta modeladora Pomada capilar Pomada fixadora Pomada modeladora Pomade Pomadas para trançar	Search type: exact result Where to search: in content Search method: act by act Gazette: Section 1 and Extra and supplementary edition	https://www.in.gov.br/leiturajornal
Internet portal of the Brazilian Health Regulatory Agency Portal (*Agência Nacional de Vigilância Sanitária* - ANVISA)	Alerts	Not applicable	Cosmetic subjects	https://antigo.anvisa.gov.br/alertas
	Data spreadsheet	Not applicable	Not applicable	https://www.gov.br/anvisa/pt-br/assuntos/cosmeticos/pomadas/pomadas-autorizadas
	News	Pomad Modela Trança	Category: Health and Sanitary Surveillance	https://www.gov.br/anvisa/pt-br/assuntos/noticias-anvisa
ANVISA electronic information system	Reports, bulletins, technical notes, letters, dispatches, memos and emails	Not applicable	Not applicable	Not applicable

The ANVISA Electronic Information System is a document and electronic administrative process management tool, which enables production, editing, signing and processing of documents and processes within the scope of ANVISA. The authors accessed documents from this system contained in administrative processes that were shared with the management division responsible for ANVISA’s cosmetovigilance actions. Unlike the other data sources, a procedure for systematic document identification and selection, such as definition and use of keywords, was not established in this case.

ANVISA provided the authors with an electronic spreadsheet in April 2024 containing the following data from its database of reported ocular adverse events related to hair styling creams: a) date of case reporting; b) date of sign and symptom onset; and c) number of cases reported to ANVISA. The spreadsheet was provided after duplicates had been eliminated, thus ensuring that only single cases were considered.

### Data extraction

An electronic spreadsheet database was created for each data source. Each selected document was coded and recorded on a row of the spreadsheet, with the characterizing variables distributed in columns. 

 The variables in the Official Gazette of the Union database included: (i) identification of the normative act; (ii) date of publication; (iii) sanitary surveillance action; and (iv) total products subject to the regulation. The ANVISA internet portal database variables included: (i) title of the news article or document; (ii) publication date; and (iii) sanitary surveillance action. The variables included in the case of the ANVISA Electronic Information System were: (i) document number; (ii) type of document; (iii) document date; and (iv) sanitary surveillance action.

After eliminating duplicate documents, the data of interest were extracted and input to the respective databases. The inclusion and exclusion criteria were then applied to complete the formation of the databases. 

### Data synthesis

Synthesis of data retrieved from eligible documents followed four steps. The first step involved understanding the information, whereby the documents were reviewed and understood in their original context. Next, thematic categories were defined within the scope of sanitary surveillance actions: marketing authorization action; cosmetovigilance action; market inspection action; and laboratory analysis action. The third stage involved classifying the documents into thematic categories, based on the subject covered in the document and the ANVISA technical division responsible for preparing it. In the case of the “news” document type, the three previous steps were carried out using Taguette software (14). The last stage involved the development of a cohesive and comprehensive narrative about ANVISA’s actions to mitigate ocular adverse events related to hair styling creams.

### Statistical analysis 

Descriptive data are presented as absolute numbers (percentages), medians and interquartile ranges. Spearman’s correlation coefficient (statistically significant when p-value<0.05) was used to verify the relationship between ANVISA’s actions and cases of ocular adverse events. The choice of this coefficient is justified because the study data do not follow a normal distribution. 

Correlation magnitude was interpreted as follows: correlation coefficients with p-value<0.40 (weak magnitude), between p-value>0.40 and p-value<0.50 (moderate) and p-value>0.50 (strong magnitude) (15).

As the data was considered as a time series, the correlation results take into account the temporal dependence of the observations. We used Gretl 2024b and Microsoft Office Excel software in the statistical analyses.

## Results

**Figure 1:**
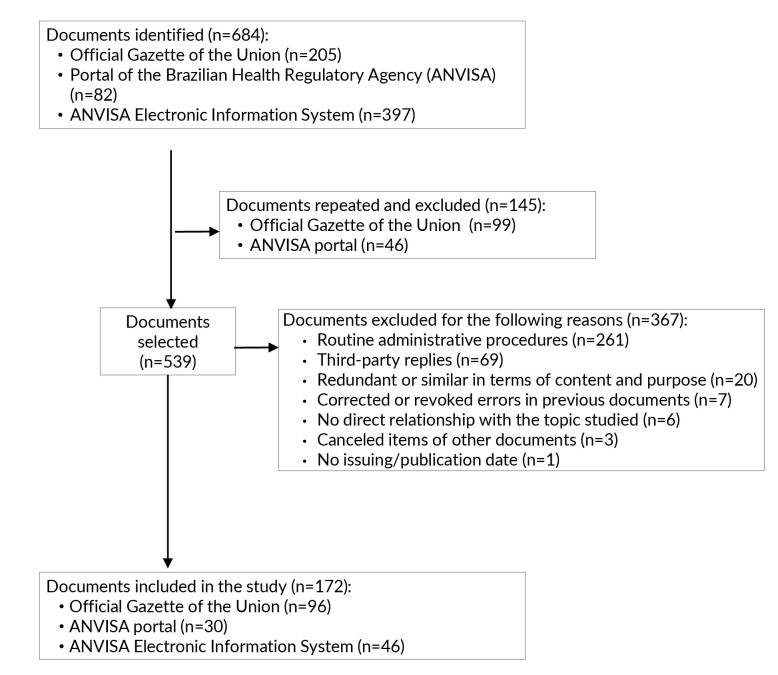
Process of identification, selection and inclusion of documents in the study

We identified 684 documents in the three data sources. Of these, 172 (25.1%) were included in the study, representing the total number of ANVISA actions (Figure 1). The five most prevalent types of documents were: normative acts (n=96; 55.8%), news articles (n=26; 15.1%), reports (n=17; 9.9%), letters (n=12; 7.0%) and emails (n=10; 5.8%). Fifteen documents were classified into more than one thematic category, with 93.3% (n=14) of these cases focused on “news”.

ANVISA coordinated its actions from its headquarters in Brasília, with the support of other bodies, such as the Ministry of Health and state and municipal health surveillance services, and the following laboratories: *Fundação Oswaldo Cruz* (Fiocruz) nuclear magnetic resonance analytical laboratory; Fiocruz National Institute of Health Quality Control; *Universidade Federal de Goiás* in vitro toxicology laboratory; and *Universidade Federal de Campina Grande* Northeast Region biomaterials laboratory.

ANVISA implemented a median of 4 actions per month (interquartile range of 7 monthly actions), between March 2022 and March 2024, to mitigate ocular adverse events. Range of variation was from 0 to 37 actions per month. It is noteworthy that, between December 2022 and March 2024 (16 months), ANVISA carried out 162 (94.2%) actions on an uninterrupted basis. The highest number of actions were recorded in February 2023 (n=37; 22.8%). 

**Figure 2:**
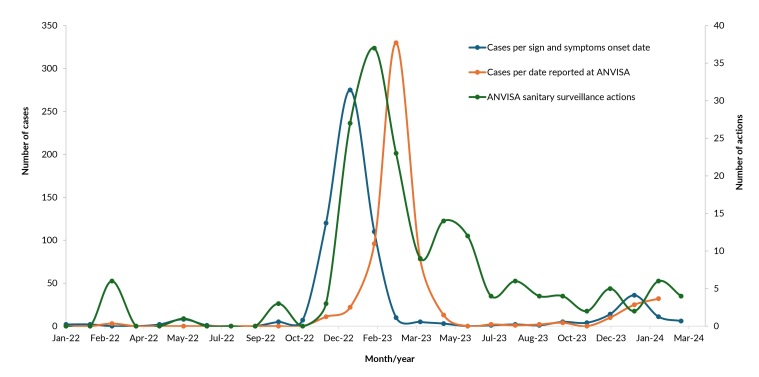
Distribution of sanitary surveillance actions implemented by the Brazilian Health Regulatory Agency (n=172) and distribution of the number of cases of ocular adverse events, by sign and symptoms onset date (n=630) and reporting date (n=636). Brazil, March 2022 to March 2024

Figure 2 shows the distribution of ANVISA actions (n=172) and the number of cases according to ocular adverse events sign and symptom onset date (n=630) and case reporting date (n=636). Three distinct periods can be seen. In the first period (March to November 2022), ANVISA’s actions were one-off (n=10). The largest number of actions was concentrated in March (n=6), coinciding with just three cases of ocular adverse events reported.

In the first period, ANVISA’s actions were: (i) prohibition of marketing, distribution, manufacture or advertising of six hair styling creams, as well as their recall, resulting from three normative acts (March 22 to October 20, 2022); (ii) cancellation of marketing authorization for four of these products, defined by ANVISA Resolution No. 1,921, dated July 10, 2022 (16); (iii) five cosmetovigilance actions, which included preparation of a report (October 25, 2022), in response to the demand received from ANVISA’s cosmetics marketing authorization area regarding “adverse reactions involving hair creams for braids”; and (iv) publication of news articles on the ANVISA internet portal, on March 23, 2022.

The content of the news article highlighted the prohibition of marketing, distribution, manufacture, advertising and use of Omegafix braid hair cream, in accordance with ANVISA Resolution No. 892, data March 22, 2022 (17). The news article also reported that “In recent days, ANVISA has become aware, via the media, of cases of eye problems reported by users after using this hair cream.”

The second period (December 2022 to June 2023), identified in Figure 2, corresponded to the largest number of ANVISA actions (n=125; 72.7%), coinciding with peaks in cases of adverse events by symptom onset date (n=515) and case reporting date (n=550). This period accounted for the highest number of cases reported to ANVISA (n=550), corresponding to 86.7% of the total in the 25 months studied. Actions intensified between January and March 2023, representing 69.6% (n=87) of total actions carried out in the period. 

Table 2 presents the main actions adopted by ANVISA to mitigate cases of adverse ocular events. The laboratory analysis actions, which began in this period, continued in the subsequent period.

**Table 2:** Ten main sanitary surveillance actions implemented by the Brazilian Health Regulatory Agency (ANVISA) in response to ocular adverse events related to hair styling creams. Brazil, December 2022 to June 2023

Data	Sanitary surveillance action	Thematic category
December 13, 2022 to January 19, 2023	ANVISA published two safety alerts: Alert No. 07/2022, about temporary blindness and other undesirable effects, supposedly caused by hair styling creams; and Alert No. 01/2023, which updated this information.	Cosmetovigilance action
December 14, 2022 to June 19, 2023	Seventeen news articles published on ANVISA’s internet portal, corresponding to 65.4% of the total news items published in the 25-month period (n=26).	Marketing authorization, cosmetovigilância, market inspection and laboratory analysis action
January 6 to June 27, 2023	ANVISA published 66 normative acts, representing 68.7% of the total during the study period (n=96). Of these, 36 were inspection measures, with the majority (69.4%) involving the suspension and recall of hair styling creams, and 30 marketing authorizations, with 86.7% referring to the cancellation of these products. Between December 2022 and June 2023, 809 styling creams had their authorizations canceled.	Inspection and marketing authorization action
January 18, 2023	Consultation restricted to the ANVISA Sentinel Hospital Network. The objective of the action was to capture records of care provided to patients with eye problems related to the use of hair styling creams in health units throughout 2022.	Cosmetovigilance action
February 4 to March 27, 2023	Field investigation carried out at the Souza Aguiar Municipal Hospital in Rio de Janeiro, with preliminary data from the second week of research conducted by the Training Program on Epidemiology Applied to the Services of the Brazilian Ministry of Health National Health System. A total of 451 patients had been treated at the health unit with ocular adverse events related to styling creams, between December 19, 2022 and January 31, 2023. Other field investigations took place in the municipality of Humaitá (Amazonas) and in the state of Pernambuco.	Cosmetovigilance action
February 10, 2023	ANVISA banned all hair styling creams, prohibiting their marketing in Brazil, through Resolution No. 475, dated February 9, 2023, due to reports of serious adverse events. This standard is part of the 66 normative acts mentioned above.	Market inspection action
February 10 to October 27, 2023	Six laboratory analysis reports were produced, including results of nuclear magnetic resonance, infrared spectrometry, physical-chemical analyses, chemical analyses, microbiological analyses and toxicological assessment of ocular irritation and corrosion due to hair styling creams. These reports were prepared by public institutions that work in partnership with ANVISA.	Laboratory analysis action
February 8 to March 23, 2023	Consultations and responses from international health authorities from ten countries plus the European Union on the occurrence of ocular adverse events related to hair styling creams. All those health authorities replied that they had had no similar cases.	Cosmetovigilance action
March 16 to April 26, 2023	Two cosmetovigilance bulletins were made available for internal consultation by ANVISA. The two documents presented, in particular, an epidemiological analysis of cases of ocular adverse events related to styling creams, in terms of time, place and person.	Cosmetovigilance action
March 20, 2023	ANVISA authorized marketing of a restricted list of hair styling creams. In the last update of this list, carried out on February 9, 2024, there were 490 hair styling creams that could be sold in Brazil.	Market inspection action and marketing authorization action

Of note is the publication of ANVISA Resolution No. 475, dated February 9, 2023 (18), which determined the precautionary ban on all hair styling creams sold in Brazil, lasting 38 days (Table 2). By February 2023, ANVISA had received reports of 132 cases of ocular adverse events. This number, however, represented 29.3% of the total number of patients treated at the Souza Aguiar Hospital Municipal, in Rio de Janeiro, with ocular adverse events related to hair styling creams, between December 19, 2022 and January 31, 2023 (n=451). 

In the third period, from July 2023 to March 2024, health surveillance actions continued despite the reduction in cases (n=83). ANVISA carried out 37 (21.5%) actions, of which 15 were marketing authorization actions, resulting in the highest number hair styling cream marketing authorization cancellations (n=3,122). This number corresponded to 79.3% of the total number of hair styling creams canceled in the 25 months studied (n=3,935).

During this period, two actions stand out that prevented future cases of adverse ocular events. The first action was the publication of ANVISA Collegiate Board Resolution No. 814, dated September 1, 2023 (19), which changed the way hair styling creams were regularized by ANVISA, changing their classification from a product subject to prior communication to a health surveillance registered product. The second action corresponded to the publication of ANVISA Resolution No. 3,566, dated September 20, 2023 (20), which prohibited marketing, distribution, manufacture and advertising of all batches of hair styling creams that were not included in the list of ANVISA authorized products. As at March 2024, there were 490 hair styling creams on the list published by ANVISA. 

The toxicology analyses obtained in this third period resulted in 6 (30.0%) samples of hair styling creams being classified as severely irritant/corrosive, out of 20 samples tested using the short time exposure cytotoxicity test method.

Our analysis of ANVISA’s actions revealed dispersion between the different thematic categories. Marketing authorization actions had the highest proportion, with 62 (39.7%). ANVISA carried out 51 (32.7%) market inspection actions, 35 (22.4%) cosmetovigilance actions and 8 (4.7%) laboratory analysis actions. Sixteen (9.4%) actions were classified in more than one thematic category, and were therefore not counted in the values ​​of each of the categories mentioned above. 

The first actions recorded in the 25 months analyzed corresponded to cosmetovigilance actions (n=4) and market inspection actions (n=1). As of January 2023, marketing authorization actions were the only thematic category that remained constant monthly until the end of the period studied.

Between January and April 2023, the period that concentrated the largest number of cases of ocular adverse events, the combination of different ANVISA actions was evident, with emphasis on the months of February to April, which recorded joint marketing authorization actions (n=22), cosmetovigilance actions (n=21), market inspection actions (n=16) and laboratory analysis actions (n=4). 

The correlation coefficients indicated a positive relationship and strong magnitude between ANVISA’s actions and the number of cases of ocular adverse events, varying between 0.53 (p-value 0.006) and 0.74 (p-value<0.001) (Table 3).

**Table 3:** Correlation coefficient between actions implemented by the Brazilian Health Regulatory Agency and the number of reports of ocular adverse events. Brazil, March 2022 to March 2024

Variables	Spearman’s coefficient	p-value
Actions as a whole and number of reports of ocular adverse events	0.74	<0.001
Marketing authorization actions and number of reports of ocular adverse events	0.64	<0.001
Market inspection actions and number of reports of ocular adverse events	0.59	0.002
Cosmetovigilance actions and number of reports of ocular adverse events	0.57	0.003
Laboratory analysis actions and number of reports of ocular adverse events	0.53	0.006

## Discussion

This study addresses an emerging public health issue related to the use of cosmetic products, specifically hair styling creams, which has been little explored. The findings indicate that ANVISA’s actions led to a reduction in cases of adverse ocular events and an improvement in the safety of hair styling creams sold in Brazil. ANVISA’s experience highlights integrated regulatory efforts focused on marketing authorization, cosmetovigilance, market inspection and laboratory analysis actions. The strong positive correlation between the number of actions and the reduction in adverse events demonstrates that the measures adopted by ANVISA were reactive to the increase in reported cases, reinforcing the importance of active surveillance and rapid interventions to control risks to public health.

This study has limitations that need to be considered when interpreting the results. The documents held on the ANVISA Electronic Information System were limited to administrative processes processed in the cosmetovigilance area. Despite efforts made to identify relevant documents, it is possible that some of them may not have been not included in the analysis. However, ANVISA’s main actions in response to ocular adverse events have been mentioned in the study. Integration with other data sources may have minimized this limitation.

The inclusion of documents encouraged by ANVISA as part of sanitary surveillance actions was controlled to avoid inflating the action count. Clear criteria were established for the inclusion and exclusion of documents, ensuring that only those with a direct impact were quantified. 

Ocular adverse events may not have been recorded on the ANVISA database, especially those of lesser severity, leading to underestimation of the real magnitude of the problem. Efforts to promote reporting, such as publishing safety alerts, may have helped to minimize this bias. The study may also be subject to selection bias, as the data were extracted from the ANVISA database, which may not reflect the context of other reporting systems, such as the Ministry of Health’s Notifiable Health Conditions Information System, which records cases of cosmetic poisoning (21). Furthermore, the descriptive nature of the analysis and the difficulty in establishing causal relationships between ANVISA’s actions and changes in reports of adverse events is another limitation of this study. 

The findings demonstrate that ANVISA adapted its interventions, according to the number of cases of ocular adverse events recorded on its information systems and the number of cases identified in field investigations that took place in the municipalities of Rio de Janeiro and Humaitá, and in the state of Pernambuco. Collecting this data helped to understand the extent of the problem, directing ANVISA’s subsequent actions. It is noteworthy that consultation with international health authorities and their informing the absence of similar events in other countries revealed a local health crisis. The publication of several news items on the ANVISA portal, including the release of three security alerts, suggests intense risk communication aimed at society, showing a strong emphasis on public awareness-raising.

Historically, in the United States, regulations have emerged in response to public health incidents, as illustrated by the case of the recall of the drug Vioxx in the 2000s (22). In relation to this incident, the importance of pharmacovigilance was highlighted, since adverse cardiovascular events were detected after the medication was used by the population (22). In Brazil, regulatory decisions are based on a normative system that aims to protect the health of the population. Just as in the North American case, many of these decisions have a reactive characteristic, which can raise criticism regarding the response time. In the case of hair styling creams, the findings indicate that the response time was reasonably timely, avoiding new serious eye injuries, by canceling marketing of products associated with adverse events. Although these reactive regulations are essential for preventing new cases, concern about response time represents a challenge, with the need for regulatory tools to be more proactive and preventive, in order to anticipate problems and avoid public health crises (22).

Hair styling cream marketing authorization only according to a restricted list and the change in the regulation model for these products, from prior communication to sanitary surveillance registration, reflect a balanced, cautious and more rigorous approach to their access. These measures also directly contributed to curbing the apparently indiscriminate availability of hair styling creams on the national market. Unauthorized and low-quality cosmetics can cause serious adverse health events, such as skin damage and other medical problems (23). In Pakistan, the wide availability of illegal cosmetics has seriously affected the population, both urban and rural, with risks of carcinogenesis and multiple organ failure (23).

Adoption of preventive measures, such as the precautionary ban on hair styling creams prior to the 2023 carnival festivities, and corrective measures, such as the withdrawal of many unsafe products from the market, helped to contain cases of adverse events in Brazil. According to the literature, cosmetics regulations are designed to prevent dangerous products from entering the market or reaching consumers, while promoting cosmetovigilance systems can reduce the occurrence of adverse health events (24).

The laboratory analyses that confirmed the relationship between hair styling creams and ocular adverse events and that justified the market inspection and marketing authorization actions took place in the third period. Some factors may have contributed to the delay in obtaining these results, and the literature points to challenges in this direction. An important aspect in preparing for health crises is the logistics involved in carrying out laboratory analyses. This includes identifying an appropriate laboratory to perform the tests, timely sample collection and transport, as well as development of sample processing protocols, validation of laboratory methods, and costs of specific tests (25-26).

The results of the correlation analysis indicate a statistically significant and positive relationship of strong magnitude between ANVISA’s actions and the number of cases of ocular adverse events. The data suggests that ANVISA implemented more actions as reports of cases of ocular adverse events increased. However, the methodological approach adopted, which is based on a descriptive and correlational study, does not allow for exploration of temporality between these two phenomena. In order to investigate the nature of these interactions, it would be necessary to apply methods that consider temporal sequence and causality, enabling a more robust analysis of the relationship between reported cases and ANVISA interventions. 

This study contributes to regulatory science and the safety of cosmetics, highlighting the role of the Brazilian National Health System (*Sistema Único de Saúde* - SUS) in sanitary surveillance actions. The data emphasize the need for an integrated and multifaceted approach in the management of adverse events related to cosmetic products. ANVISA’s actions exemplify this strategy, by combining strict regulation, active inspection, continuous cosmetovigilance, laboratory analyses and public awareness-raising. In the context of the SUS, it is important that health services improve cosmetovigilance actions, in order to strengthen the monitoring system and achieve more robust assessment, enabling the systematic collection of data on adverse events and analysis of their causes. This study can serve as a model for other regulatory agencies and guide future regulatory measures that ensure consumer safety.

## Data Availability

The authors have made the study database available in an electronic spreadsheet containing data related to the sources Diário Oficial da União and Portal da Anvisa, at the following address: https://doi.org/10.17605/osf.io/edw5x
