# Supplementary material for: Actions to mitigate ocular adverse events related to hair styling cosmetics in Brazil: a descriptive and correlational study (2022-2024)
Source: Epidemiol Serv Saude. 2025 May 2;34:e20240449. doi: 10.1590/S2237-96222024v34e20240449.en (PMC12052321; doi:10.1590/S2237-96222024v34e20240449.en)
Supplement: Translation

# Translation: Ações de mitigação de eventos adversos oculares relacionados a cosméticos para modelar cabelos no Brasil: estudo descritivo e correlacional (2022-2024)

**DOI:** 10.1590/S2237-96222024v34e20240449.pt

**Authors:** Daniel Marques Mota, Leonardo Oliveira Leitão, Ronald Santos Silva, Cássia de Fátima Rangel Fernandes, Marcus Tolentino Silva

**Affiliations:** 1 Agência Nacional de Vigilância Sanitária, Brasília, DF, Brasil; 2 Instituto Nacional de Controle de Qualidade em Saúde, Rio de Janeiro, RJ, Brasil; 3 Universidade de Brasília, Brasília, DF, Brasil

**Keywords:** Preparações para Cabelo, Lesões da Córnea, Vigilância de Produtos Comercializados, Análise Documental, Correlação de Dados

## Abstract

**Objetivo:**

Descrever as ações de vigilância sanitária implementadas pela Agência Nacional de Vigilância Sanitária (Anvisa) para mitigar casos de eventos adversos oculares relacionados a pomadas modeladoras no Brasil e verificar a correlação entre os casos relatados e essas ações.

**Métodos:**

Estudo descritivo e correlacional a partir de análise de documentos e do banco de notificações da Anvisa, entre março de 2022 e março de 2024. Utilizou-se o coeficiente de correlação de Spearman para examinar a correlação entre as ações da Anvisa e os casos de eventos adversos (p-valor<0,05).

**Resultados:**

Identificaram-se três períodos distintos em relação às ações da Anvisa (n=172) e aos casos relatados (n=636). O segundo período (dezembro de 2022 a junho de 2023) apresentou o maior número de ações (n=125; 72,7%), coincidindo com o pico de notificações dos casos (n=550). Nesse período, foram publicados dois alertas de segurança e determinada a interdição cautelar de todas as pomadas modeladoras comercializadas no país, que durou 38 dias. No terceiro período (julho de 2023 a março de 2024), ocorreu o maior número de cancelamentos de comercialização de pomadas (n=3.122; 79,3%), e análises toxicológicas indicaram que seis (30,0%) amostras foram classificadas como irritante severo/corrosivo. Houve correlação positiva e forte entre as ações e os casos relatados, variando de 0,53 (p-valor 0,006) a 0,74 (p-valor<0,001).

**Conclusão:**

A forte correlação positiva demonstra que as ações da Anvisa foram reativas à elevação das notificações dos casos, reforçando a importância de vigilância ativa e intervenções rápidas para controlar os riscos à saúde pública.

## Introdução

A segurança dos cosméticos intensificou-se, nos últimos anos, impulsionada pelo aumento do consumo desses produtos (1), desenvolvimento de regulamentações rigorosas (2), atualizações de ingredientes químicos proibidos em cosméticos (3) e registros de eventos adversos que afetam a qualidade de vida dos consumidores e sobrecarregam os sistemas de saúde (4-6). 

Os cosméticos, em sua maioria, estão isentos de avaliação regulatória prévia pela autoridade sanitária antes de serem disponibilizados no mercado (7), cabendo à empresa garantir que sejam seguros à saúde. Essa simplificação regulatória também evidencia a necessidade de vigilância pós-comercialização contínua e rigorosa para proteger a população (7).

A vigilância da segurança dos cosméticos na pós-comercialização é conhecida como cosmetovigilância. Desde 2005, no Brasil, as empresas fabricantes de cosméticos são obrigadas por norma a implementarem sistema de cosmetovigilância e relatarem situações que impliquem risco para a saúde à Agência Nacional de Vigilância Sanitária (Anvisa) (8). Outros países têm seguido essa tendência, exigindo que empresas notifiquem às autoridades sanitárias eventos adversos graves relacionados a cosméticos (2). 

A partir de março de 2022, no Brasil, os cosméticos para modelar cabelos, denominados pomadas modeladoras, passaram a ser associados a riscos à saúde ocular, evidenciados por relatos de ardência, dor, lacrimejamento, irritação, hiperemia e, em casos extremos, queimadura da córnea e cegueira temporária (9-10). Os casos aumentaram em dezembro de 2022 (11), tornando-se uma crise sanitária. 

Este estudo objetiva descrever as ações de vigilância sanitária implementadas pela Anvisa para mitigar casos de eventos adversos oculares relacionados a pomadas modeladoras no Brasil e verificar a correlação entre os casos relatados e essas ações. 

## Métodos

### Desenho do estudo

Estudo descritivo e correlacional, realizado a partir da análise de documentos produzidos ou geridos pela Anvisa, incluindo banco das notificações dos casos de eventos adversos oculares relacionados a pomadas modeladoras. O estudo compreendeu o período de 1º de março de 2022 a 31 de março de 2024. O conhecimento tácito (12) de um dos autores sobre a atuação da Anvisa orientou a delimitação do período, definição de palavras-chave e fontes de documentos. 

### Contexto

Este estudo baseou-se em ações de vigilância sanitária executadas pela Anvisa, que regula e monitora os cosméticos para garantir sua segurança e eficácia no mercado brasileiro (13). A Anvisa foi selecionada como foco do estudo pelo papel central na regulação de cosméticos no Brasil, permitindo implementar e monitorar ações de enfrentamento a crises sanitárias; e pela produção de acervo documental, que oferece visão detalhada das ações adotadas para mitigar eventos adversos relacionados a cosméticos. 

### Definição de ação de vigilância sanitária

Uma “ação de vigilância sanitária” foi definida como a produção de documentos emitidos pela Anvisa ou por outras entidades, cuja criação foi diretamente estimulada pela Agência em decorrência dos eventos adversos oculares relacionados a pomadas modeladoras. Esses documentos incluíram relatórios de investigação de campo e de análises laboratoriais e e-mails de autoridades sanitárias internacionais. Esta abordagem baseou-se na premissa de que esses documentos operacionalizaram as ações de vigilância sanitária da Anvisa. 

### Critérios de inclusão e exclusão

Os critérios de inclusão foram: (i) documentos que contribuíram para o objetivo do estudo, como atos normativos, alertas, planilhas de dados, notícias, boletins, relatórios, ofícios e e-mails; (ii) documentos sobre medidas de fiscalização do mercado, procedimentos de autorização de comercialização, ações de cosmetovigilância, incluindo investigação de campo, e análises laboratoriais; e (iii) documentos gerados entre março de 2022 e março de 2024. 

Os critérios de exclusão contemplaram: (i) documentos que retificaram ou revogaram erros de documentos anteriores; (ii) documentos que anularam itens de outros documentos sem fornecer novas informações; (iii) documentos sem relação direta com o tema; (iv) documentos redundantes ou semelhantes, em que apenas o primeiro ou o mais completo foi incluído, a menos que os subsequentes tenham apresentado novas informações relevantes; (v) documentos sem data de elaboração; (vi) documentos de procedimentos administrativos sem relevância para o estudo; e (vii) documentos de resposta de terceiros irrelevantes ao estudo.

### Coleta dos dados

As fontes de dados foram: Diário Oficial da União do Poder Executivo; Portal eletrônico da Anvisa; e Sistema Eletrônico de Informações da Anvisa. A coleta dos dados ocorreu entre julho e agosto de 2024 (Tabela 1). 

**Tabela 1:** Fontes de dados, tipos de documentos e estratégias de buscas dos documentos utilizadas neste estudo

Fonte de dados	Tipo de documento	Palavras-chave	Filtros de seleção	Endereço eletrônico na internet
Diário Oficial da União	Atos normativos	Cera fixadora Cera modeladora Gel cera Gel cola Intoxicação ocular Pasta fixadora Pasta modeladora Pomada capilar Pomada fixadora Pomada modeladora Pomade Pomadas para trançar	Tipo de pesquisa: resultado exato Onde pesquisar: no conteúdo Forma de pesquisa: ato a ato Jornal: Seção 1 e Edição extra e suplementar	https://www.in.gov.br/leiturajornal
Portal eletrônico da Agência Nacional de Vigilância Sanitária (Anvisa)	Alertas	Não se aplica	Assuntos: cosméticos	https://antigo.anvisa.gov.br/alertas
	Planilha de dados	Não se aplica	Não se aplica	https://www.gov.br/anvisa/pt-br/assuntos/cosmeticos/pomadas/pomadas-autorizadas
	Notícias	Pomad Modela Trança	Categoria: Saúde e Vigilância Sanitária	https://www.gov.br/anvisa/pt-br/assuntos/noticias-anvisa
Sistema Eletrônico de Informações da Anvisa	Relatórios, boletins, notas técnicas, ofícios, despachos, memorandos e e-mails	Não se aplica	Não se aplica	Não se aplica

O Sistema Eletrônico de Informações é uma ferramenta de gestão de documentos e processos administrativos eletrônicos, que permite a produção, edição, assinatura e tramitação de documentos e processos no âmbito da Anvisa. Os autores acessaram documentos desse sistema contidos em processos administrativos que foram compartilhados com a gerência responsável pelas ações de cosmetovigilância da Anvisa. Diferentemente das outras fontes de dados, não foi estabelecida identificação e seleção sistematizadas de documentos, como definição e uso de palavras-chave. 

Os dados do banco das notificações dos eventos adversos oculares relacionados a pomadas modeladoras, fornecido em planilha eletrônica pela Anvisa em abril de 2024, foram: a) data da notificação do caso; b) data de início dos sinais e sintomas; e c) número de casos notificados à Anvisa. A planilha foi disponibilizada após a eliminação de duplicatas, garantindo que apenas casos únicos fossem considerados.

### Extração dos dados

Um banco de dados, em planilha de eletrônica, foi elaborado para cada fonte de dados. Cada documento selecionado foi codificado e registrado em uma linha da planilha, com as variáveis caracterizadoras distribuídas em colunas. 

 As variáveis do banco de dados do Diário Oficial da União incluíram: (i) identificação do ato normativo; (ii) data de publicação; (iii) ação de vigilância sanitária; e (iv) total de produtos sujeitos ao regulamento. As variáveis do banco do portal eletrônico contemplaram: (i) título da notícia ou do documento; (ii) data de publicação; e (iii) ação de vigilância sanitária. Para o Sistema Eletrônico de Informações, as variáveis incluídas foram: (i) número do documento; (ii) tipo de documento; (iii) data do documento; e (iv) ação de vigilância sanitária.

Após a eliminação dos documentos repetidos, os dados de interesse foram extraídos e inseridos nos respectivos bancos de dados. Em seguida, aplicaram-se os critérios de inclusão e exclusão para concluir a formação dos bancos de dados. 

### Síntese dos dados

A síntese dos dados dos documentos elegíveis seguiu quatro etapas. Primeiramente, houve a compreensão das informações, onde os documentos foram revisados e entendidos em seu contexto original. Em seguida, definiram-se categorias temáticas no âmbito das ações de vigilância sanitária: ação de autorização de comercialização; ação de cosmetovigilância; ação de fiscalização do mercado; e ação de análise laboratorial. A terceira etapa envolveu a classificação dos documentos nas categorias temáticas, baseada no assunto abordado no documento e na área técnica da Anvisa responsável pela elaboração. Para o tipo de documento “notícias”, as três etapas anteriores foram realizadas com utilização do software Taguette (14). A última etapa envolveu a elaboração de narrativa coesa e abrangente sobre as ações da Anvisa para mitigar eventos adversos oculares relacionados a pomadas modeladoras.

### Análise estatística

Os dados descritivos são apresentados como número absoluto (porcentagem), mediana e intervalo interquartil. O coeficiente de correlação de Spearman, com nível de significância estatística de p-valor<0,05, foi utilizado para verificar a relação entre as ações da Anvisa e os casos de eventos adversos oculares. A escolha desse coeficiente se justifica em razão de os dados do estudo não obedecerem à distribuição normal. 

A magnitude das correlações foi interpretada da seguinte forma: coeficientes de correlação p-valor<0,40 (fraca magnitude), entre p-valor>0,40 e p-valor<0,50 (moderada) e p-valor>0,50 (forte magnitude) (15).

Em decorrência de os dados terem sido considerados como série temporal, os resultados da correlação levam em conta a dependência temporal das observações. Nas análises estatísticas, foi utilizado o software Gretl 2024b e Microsoft Office Excel.

## Resultados

Identificaram-se 684 documentos nas três fontes de dados. Destes, 172 (25,1%) foram incluídos no estudo, representando o total das ações da Anvisa (Figura 1). Os cinco tipos de documentos mais prevalentes foram: atos normativos (n=96; 55,8%), notícias (n=26; 15,1%), relatórios (n=17; 9,9%), ofícios (n=12; 7,0%) e e-mails (n=10; 5,8%). Quinze documentos foram classificados em mais de uma categoria temática, sendo que 93,3% (n=14) desses casos se concentraram em “notícias”. 

**Figura 1:** Processo de identificação, seleção e inclusão dos documentos no estudo

A Anvisa coordenou suas ações a partir de sua sede em Brasília, com apoio de outros órgãos, como o Ministério da Saúde e vigilâncias sanitárias estaduais e municipais, e dos seguintes laboratórios: laboratório analítico de ressonância magnética nuclear da Fundação Oswaldo Cruz, Instituto Nacional de Controle de Qualidade em Saúde da Fiocruz, laboratório de toxicologia in vitro da Universidade Federal de Goiás e laboratório de biomateriais do Nordeste da Universidade Federal de Campina Grande.

A Anvisa implementou mediana de 4 ações por mês (intervalo interquartil de 7 ações mensais), entre março de 2022 e março de 2024, para mitigar os eventos adversos oculares. A amplitude da variação foi de 0 a 37 ações por mês. Destaca-se que, entre dezembro de 2022 e março de 2024 (16 meses), 162 (94,2%) ações foram realizadas pela Anvisa de forma ininterrupta. Fevereiro de 2023 registrou o maior número de ações (n=37; 22,8%). 

A Figura 2 apresenta a distribuição das ações da Anvisa (n=172) e o número de casos de eventos adversos oculares por data de início dos sinais e sintomas (n=630) e data de notificação (n=636). É possível notar a presença de três períodos. No primeiro período (março a novembro de 2022), as ações da Anvisa foram pontuais (n=10). Março concentrou o maior número de ações (n=6), coincidindo com a notificação dos três únicos casos de eventos adversos oculares.

**Figura 2:** Distribuição das ações de vigilância sanitária implementadas pela Agência Nacional de Vigilância Sanitária (n=172) e do número de casos de eventos adversos oculares, por data de início dos sinais e sintomas (n=630) e data de notificação (n=636). Brasil, março de 2022 a março de 2024

No primeiro período, as ações da Anvisa foram: (i) proibição da comercialização, distribuição, fabricação, propaganda e recolhimento de seis pomadas modeladoras, resultante de três atos normativos (22 de março a 20 de outubro de 2022); (ii) cancelamento de autorização de comercialização de quatro desses produtos, definido pela Resolução nº 1.921, de 10 de julho de 2022 (16); (iii) cinco ações de cosmetovigilância, que incluiu a elaboração de relatório (25 de outubro de 2022), o qual respondia a demanda da área de autorização de comercialização de cosméticos sobre “reações adversas envolvendo pomadas para tranças”; e (iv) publicação de notícia no portal eletrônico da Anvisa, em 23 de março de 2022.

O conteúdo da notícia destacou a proibição da comercialização, distribuição, fabricação, propaganda e uso da pomada para tranças Ômegafix, por meio da Resolução nº 892, de 22 de março de 2022 (17). A notícia também informou que “Nos últimos dias, a Agência tomou conhecimento, pela mídia, de casos sobre problemas oculares relatados por usuários após o uso da pomada.”

O segundo período (dezembro de 2022 a junho de 2023) identificado na Figura 2 correspondeu ao maior número de ações da Anvisa (n=125; 72,7%), coincidindo com os picos dos casos de eventos adversos por data de início dos sintomas (n=515) e data de notificação (n=550). Este período registrou o maior número de casos notificados à Anvisa (n=550), correspondendo a 86,7% do total nos 25 meses estudados. As ações se intensificaram entre janeiro e março de 2023, representando 69,6% (n=87) do montante de ações realizadas no período. 

A Tabela 2 apresenta as principais ações adotadas pela Anvisa para mitigar os casos de eventos adversos oculares. As ações de análise laboratorial, que se iniciaram neste período, tiveram continuidade no período subsequente.

**Tabela 2:** Dez principais ações de vigilância sanitária implementadas pela Agência Nacional de Vigilância Sanitária (Anvisa) em resposta aos eventos adversos oculares relacionados a pomadas modeladoras. Brasil, dezembro de 2022 a junho de 2023

Data	Ação de vigilância sanitária	Categoria temática
13 de dezembro de 2022 a 19 de janeiro de 2023	A Anvisa publicou dois alertas de segurança: Alerta nº 07/2022, sobre cegueira temporária e outros efeitos indesejáveis, supostamente ocasionados por pomadas modeladoras; e Alerta nº 01/2023, que atualizou essas informações.	Ação de cosmetovigilância
14 de dezembro de 2022 a 19 de junho de 2023	Dezessete notícias publicadas no portal eletrônico da Anvisa, correspondendo a 65,4% do total de notícias publicadas no período de 25 meses (n=26).	Ação de autorização de comercialização, cosmetovigilância, fiscalização do mercado e análise laboratorial
6 de janeiro a 27 de junho de 2023	A Anvisa publicou 66 atos normativos, representando 68,7% do total no período do estudo (n=96). Destes, 36 foram medidas de fiscalização, com a maioria (69,4%) envolvendo a suspensão e recolhimento de pomadas modeladoras, e 30 autorizações de comercialização, com 86,7% referentes ao cancelamento desses produtos. Entre dezembro de 2022 e junho de 2023, 809 pomadas modeladoras tiveram suas autorizações canceladas.	Ação de fiscalização e autorização de comercialização
18 de janeiro de 2023	Consulta restrita à Rede de Hospitais Sentinela da Anvisa. O objetivo da ação foi captar registros de atendimentos de pacientes com problemas oculares relacionados ao uso de pomadas modeladoras de cabelos em unidades de saúde ao longo de 2022.	Ação de cosmetovigilância
4 de fevereiro a 27 de março de 2023	Investigação de campo realizada no Hospital Municipal Souza Aguiar do Rio de Janeiro, com dados preliminares da segunda semana de pesquisa conduzida pelo Programa de Treinamento em Epidemiologia Aplicada aos Serviços do Sistema Único de Saúde do Ministério da Saúde. Um total de 451 pacientes haviam sido atendidos na unidade de saúde com eventos adversos oculares relacionados a pomadas modeladoras, entre 19 de dezembro de 2022 e 31 de janeiro de 2023. Outras investigações de campo ocorreram no município de Humaitá (Amazonas) e no estado de Pernambuco.	Ação de cosmetovigilância
10 de fevereiro de 2023	A Anvisa interditou todas as pomadas modeladoras de cabelos, proibindo sua comercialização no país, por meio da Resolução nº 475, de 9 de fevereiro de 2023, devido a relatos de eventos adversos grave. Esta norma integra os 66 atos normativos referidos anteriormente.	Ação de fiscalização do mercado
10 de fevereiro a 27 de outubro de 2023	Seis relatórios de análises laboratoriais foram produzidos, incluindo resultados de ressonância magnética nuclear, espectrometria de infravermelho, análises físico-químicas, químicas, microbiológicas e avaliação toxicológica de irritação e corrosão ocular de pomadas modeladoras. Esses relatórios foram elaborados por instituições públicas parceiras da Anvisa.	Ação de análise laboratorial
8 de fevereiro a 23 de março de 2023	Consultas e respostas de autoridades sanitárias internacionais de dez países e mais a União Europeia sobre a ocorrência de eventos adversos oculares relacionados a pomadas modeladoras. Houve a negativa de casos semelhantes por parte de todas as autoridades sanitárias.	Ação de cosmetovigilância
16 de março a 26 de abril de 2023	Dois boletins de cosmetovigilância foram disponibilizados para consulta interna da Anvisa. Os dois documentos apresentaram, em especial, uma análise epidemiológica dos casos de eventos adversos oculares relacionados a pomadas modeladoras, em termos de tempo, lugar e pessoa.	Ação de cosmetovigilância
20 de março de 2023	A Anvisa autoriza a comercialização de uma lista restrita de pomadas modeladoras. Na última atualização dessa lista, realizada em 9 de fevereiro de 2024, constavam 490 pomadas modeladoras passíveis de comercialização no país.	Ação de fiscalização do mercado e autorização de comercialização

Destaca-se a publicação da Resolução nº 475, de 9 de fevereiro de 2023 (18), que determinou a interdição cautelar de todas as pomadas modeladoras comercializadas no país, com duração de 38 dias (Tabela 2). Até fevereiro de 2023, a Anvisa havia recebido a notificação de 132 casos de eventos adversos oculares. Este número, no entanto, representava 29,3% do total de pacientes atendidos no Hospital Municipal Souza Aguiar, no Rio de Janeiro, com eventos adversos oculares relacionados a pomadas modeladoras, entre 19 de dezembro de 2022 e 31 de janeiro de 2023 (n=451). 

No terceiro período, de julho de 2023 a março de 2024, apesar da redução dos casos (n=83), as ações de vigilância sanitária continuaram. A Anvisa realizou 37 (21,5%) ações, das quais 15 foram ações de autorização de comercialização, resultando no maior número de cancelamentos de pomadas modeladoras (n=3.122). Este quantitativo correspondeu a 79,3% do total de pomadas cancelados nos 25 meses estudados (n=3.935).

Destacam-se, neste período, duas ações que evitaram futuros casos de eventos adversos oculares. A primeira ação foi a publicação da Resolução da Diretoria Colegiada nº 814, de 1º de setembro de 2023 (19), que alterou a forma de regularização das pomadas modeladoras junto à Anvisa, mudando a classificação de produto sujeito à comunicação prévia para produto sob registro sanitário. A segunda ação correspondeu à publicação da Resolução nº 3.566, de 20 de setembro de 2023 (20), que proibiu a comercialização, distribuição, fabricação e propaganda de todos os lotes de pomadas modeladoras que não estivessem incluídas na lista de produtos autorizados pela Anvisa. Até março de 2024, havia 490 pomadas modeladoras na lista publicada pela Anvisa. 

As análises toxicológicas, obtidas neste terceiro período, resultaram no enquadramento de 6 (30,0%) amostras de pomadas modeladoras na categoria de irritante severo/corrosivo, de 20 amostras testadas pelo método de citotoxicidade de curta exposição.

A análise das ações da Anvisa revelou dispersão entre as diferentes categorias temáticas. As ações de autorização de comercialização apresentaram a maior proporção, com 62 (39,7%). A Anvisa realizou 51 (32,7%) ações de fiscalização do mercado, 35 (22,4%) de cosmetovigilância e 8 (4,7%) ações de análise laboratorial. Dezesseis (9,4%) ações foram classificadas em mais de uma categoria temática, não sendo contabilizadas nos valores de cada uma das categorias referidas anteriormente. 

As primeiras ações registradas nos 25 meses analisados corresponderam às ações de cosmetovigilância (n=4) e de fiscalização do mercado (n=1). A partir de janeiro de 2023, as ações de autorização de comercialização foram a única categoria temática que se manteve constante mensalmente até o fim do período estudado.

Entre janeiro e abril de 2023, período que concentrou o maior número de casos de eventos adversos oculares, evidenciou-se a combinação das diferentes ações da Anvisa, com destaque para os meses de fevereiro a abril, que registraram as ações conjuntas de autorização de comercialização (n=22), cosmetovigilância (n=21), fiscalização do mercado (n=16) e análise laboratorial (n=4). 

Os coeficientes de correlação indicaram relação positiva e forte magnitude entre as ações da Anvisa e o número de casos de eventos adversos oculares, variando entre 0,53 (p-valor 0,006) e 0,74 (p-valor<0,001) (Tabela 3).

**Tabela 3:** Coeficiente de correlação entre ações implementadas pela Agência Nacional de Vigilância Sanitária e o número de notificações de eventos adversos oculares. Brasil, março de 2022 a março de 2024

Variáveis	Coeficiente de Spearman	p-valor
Conjunto de ações e número de notificações de eventos adversos oculares	0,74	<0,001
Ação de autorização de comercialização e número de notificações de eventos adversos oculares	0,64	<0,001
Ação de fiscalização do mercado e número de notificações de eventos adversos oculares	0,59	0,002
Ação de cosmetovigilância e número de notificações de eventos adversos oculares	0,57	0,003
Ação de análise laboratorial e número de notificações de eventos adversos oculares	0,53	0,006

## Discussão

O estudo aborda uma questão de saúde pública emergente relacionada ao uso de produtos cosméticos, especificamente pomadas modeladoras, pouco explorada. Os achados indicam que as ações da Anvisa levaram à redução nos casos de eventos adversos oculares e à melhoria na segurança das pomadas comercializadas no país. A experiência da Anvisa evidencia os esforços regulatórios integrados e concentrados em ações de autorização de comercialização, cosmetovigilância, fiscalização do mercado e análise laboratorial. A forte correlação positiva entre o número de ações e o aumento dos eventos adversos demonstra que as medidas adotadas pela Anvisa foram reativas à elevação dos casos relatados, reforçando a importância de vigilância ativa e intervenções rápidas para controlar riscos à saúde pública.

Este estudo apresenta limitações que devem ser consideradas, ao serem interpretados os resultados. Os documentos do Sistema Eletrônico de Informações foram limitados a processos administrativos tramitados à área de cosmetovigilância. Apesar dos esforços para identificar documentos relevantes, é possível que alguns deles não tenham sido incluídos na análise. No entanto, as principais ações da Anvisa em resposta aos eventos adversos oculares foram mencionadas no estudo. A integração com outras fontes de dados pode ter minimizado essa limitação.

A inclusão de documentos estimulados pela Anvisa como parte das ações de vigilância sanitária foi controlada para evitar inflacionar a contagem de ações. Estabeleceram-se critérios claros para a inclusão e exclusão de documentos, garantindo que apenas aqueles com impacto direto fossem quantificados. 

Eventos adversos oculares podem não ter sido registrados no banco de dados da Anvisa, especialmente os de menor gravidade, levando à subestimação da real magnitude do problema. Esforços para promover a notificação, como a publicação de alertas de segurança, podem ter contribuído para minimizar esse viés. O estudo, também, pode estar sujeito ao viés de seleção, pois os dados foram extraídos de banco da Anvisa, que pode não refletir o contexto de outros sistemas de notificação, a exemplo do Sistema de Informação de Agravos de Notificação do Ministério da Saúde, que registra casos de intoxicações por cosméticos (21). Além disso, a natureza descritiva da análise e a dificuldade em estabelecer relações causais entre as ações da Anvisa e as mudanças nas notificações de eventos adversos é outra limitação do estudo. 

Os achados demonstram que a Anvisa adaptou suas intervenções, conforme o número de casos de eventos adversos oculares registrado em seus sistemas de informação e o número de casos identificados em investigações de campo que ocorreram nos municípios do Rio de Janeiro e de Humaitá, e em Pernambuco. A coleta desses dados ajudou a entender a extensão do problema, direcionando as ações subsequentes da Anvisa. Destaca-se que a consulta com autoridades sanitárias internacionais e a negativa de eventos similares em outros países revelaram uma crise sanitária local. A publicação de várias notícias no portal da Anvisa, incluindo a divulgação de três alertas de segurança, sugere uma comunicação do risco intensa dirigida à sociedade, mostrando forte ênfase na conscientização pública.

Historicamente, nos Estados Unidos, as regulamentações surgiram em resposta a incidentes de saúde pública, como ilustrado pelo caso da retirada do medicamento Vioxx nos anos 2000 (22). Em relação a esse incidente, destacou-se a importância da farmacovigilância, uma vez que os eventos cardiovasculares adversos foram detectados após o medicamento ser utilizado pela população (22). No Brasil, as decisões regulatórias são fundamentadas por sistema normativo que objetiva proteger a saúde da população. Da mesma forma que no caso norte-americano, muitas dessas decisões têm característica reativa, o que pode suscitar críticas quanto ao tempo de resposta. No caso das pomadas modeladoras, os achados indicam que o tempo de resposta foi razoavelmente oportuno, evitando novas lesões oculares graves, por meio do cancelamento da comercialização dos produtos associados aos eventos adversos. Embora essas regulamentações reativas sejam essenciais para evitar novos casos, a preocupação com o tempo de resposta reflete um desafio, havendo necessidade de que as ferramentas regulatórias se mostrem mais proativas e preventivas, de forma a antecipar problemas e evitar crises de saúde pública (22).

A autorização de comercialização de pomadas modeladoras em lista restrita e a mudança no modelo de regulação desses produtos, de comunicação prévia para registro sanitário, refletem uma abordagem equilibrada, cautelosa e mais rigorosa para o acesso a eles. Essas medidas também contribuíram diretamente para frear a disponibilidade aparentemente indiscriminada das pomadas modeladoras no mercado nacional. Cosméticos não autorizados e de baixa qualidade podem causar sérios eventos adversos à saúde, como danos na pele e outros problemas médicos (23). No Paquistão, a ampla disponibilidade de cosméticos ilegais tem afetado gravemente a população, tanto urbana quanto rural, com riscos de carcinogênese e falência de múltiplos órgãos (23). 

A adoção de medidas preventivas, como a interdição cautelar das pomadas modeladoras anterior às festas carnavalescas de 2023, e corretivas, como a retirada de muitos produtos inseguros do mercado, ajudaram a conter os casos de eventos adversos no país. De acordo com a literatura, os regulamentos de cosméticos são concebidos para evitar que produtos perigosos entrem no mercado ou cheguem aos consumidores, enquanto a promoção de sistemas de cosmetovigilância pode reduzir a ocorrência de eventos adversos à saúde (24).

As análises laboratoriais que confirmaram a relação de pomadas modeladoras com os eventos adversos oculares e que respaldaram as ações de fiscalização do mercado e de autorização de comercialização foram alcançadas no terceiro período. Alguns fatores podem ter contribuído para a demora na obtenção desses resultados, e a literatura aponta desafios nessa direção. Um aspecto importante na preparação para crises sanitárias é a logística envolvida na realização das análises laboratoriais. Isso inclui identificar um laboratório apropriado para realizar os testes, coleta, envio e desenvolvimento de protocolos de processamento de amostras oportunos, validação de métodos laboratoriais e custos de testes específicos (25-26).

Os resultados da análise de correlação indicam relação estatisticamente significativa e positiva de forte magnitude entre as ações da Anvisa e o número de casos de eventos adversos oculares. Os dados sugerem que, à medida que as notificações de casos de eventos adversos oculares aumentaram, a Anvisa implementou mais ações. No entanto, a abordagem metodológica adotada, que se baseia em estudo descritivo e correlacional, não permite uma exploração da temporalidade entre esses dois fenômenos. Para investigar a natureza dessas interações, seria necessário aplicar métodos que considerassem a sequência temporal e a causalidade, possibilitando análise mais robusta da relação entre as notificações e as intervenções da Anvisa. 

Este estudo contribui para a ciência regulatória e a segurança dos cosméticos, destacando a atuação do Sistema Único de Saúde (SUS) nas ações de vigilância sanitária. Os dados enfatizam a necessidade de abordagem integrada e multifacetada na gestão de eventos adversos relacionados a produtos cosméticos. A atuação da Anvisa exemplifica essa estratégia, ao combinar regulamentação rigorosa, fiscalização ativa, cosmetovigilância contínua, análises laboratoriais e conscientização da população. No contexto do SUS, é importante que os serviços de saúde aprimorem ações de cosmetovigilância, a fim de fortalecer o sistema de monitoramento e uma avaliação mais robusta, possibilitando coleta sistemática de dados sobre eventos adversos e análise de suas causas. O estudo pode servir como modelo para outras agências reguladoras e direcionar futuras medidas regulatórias que garantam a segurança dos consumidores.

## Data Availability

Os autores disponibilizaram o banco de dados em planilha eletrônica contendo os dados relacionados às fontes Diário Oficial da União e Portal da Anvisa, no seguinte endereço: https://doi.org/10.17605/osf.io/edw5x
